# Patient Caregiver Perspectives on Accessing Language Interpretation in a Pediatric Emergency Department

**DOI:** 10.1089/heq.2024.0010

**Published:** 2024-09-12

**Authors:** Emily A. Hartford, Nicholas Dimenstein, Dwight Barry, K. Casey Lion

**Affiliations:** ^1^ Division of Emergency Medicine, Department of Pediatrics, Seattle Children’s Hospital, University of Washington, Seattle, Washington, USA.; ^2^ Division of Emergency Medicine, Children’s National Hospital, George Washington University School of Medicine and Health Sciences, Washington, District of Columbia, USA.; ^3^ Clinical Analytics, Seattle Children’s Hospital, Seattle, Washington, USA.; ^4^ Division of General Pediatrics; and Seattle Children’s Research Institute, Department of Pediatrics, Center for Child Health, Behavior, and Development, University of Washington School of Medicine, Seattle, Washington, USA.

**Keywords:** health equity, interpretation, emergency care

## Abstract

Patients and caregivers with a language for care other than English (LOE) are at risk for inequitable care in the pediatric emergency department (ED). Professional interpretation (PI) improves outcomes, but there can be complexity in determining optimal language for care and interpretation need. Our goal was to learn more about the perspectives from caregivers who speak different languages regarding interpretation with a survey near ED discharge. Caregivers of patients with LOE, identified during ED check-in, were approached by research staff using PI near ED discharge. A survey was completed via interpreter or bilingual research staff prior to discharge or by phone within 48 h. Answers were entered into REDCap and analyzed descriptively. A total of 154 participants were approached; 49 completed a survey between April and November 2021. A variety of languages were spoken in the sample (*n* = 15) and represented the ED population. Twenty percent of caregivers with LOE also reported good comprehension in English. Families indicated a desire for interpretation at various stages of the ED encounter, reported different interpretation needs among family members, and indicated interest in family-initiated interpreter access. Determining optimal language for care and provision of PI during ED encounters can be complex. In this study, we report caregiver perspectives on the use of PI. Most participants wanted PI at all stages of the ED visit and were interested in accessing it themselves. Future directions are to pilot family-initiated access to PI to tailor its use to the needs of patients and families.

## Introduction

Patients and caregivers who use a language other than English for care (LOE) are at risk for inequitable health care and poor outcomes. In the pediatric emergency department (PED) these inequities include missed diagnoses, longer wait times, a higher risk of unplanned return visits, adverse events, and medication errors.^1–7^ Patients and families with LOE also report more communications challenges, prescription errors, and lower trust for their care teams.^1,8^ Professional interpretation improves outcomes for patients with LOE,^9^ but it is often underutilized in clinical settings.^7,10–12^

Accurately determining a patient and their family’s language for care can be difficult. Asking about language preference alone may not correlate well with language comprehension in a medical setting.^13^ This is particularly challenging for pediatric patients where multiple caregivers may have differing language preferences. The term “limited English proficiency” is from a question in the United States Census where participants self-rank their level of English proficiency and is often utilized; however, it does not accurately reflect the language questions typically used in medical settings. The provision of medical interpretation is required by law and recommended by national health care standards,^14^ but knowing the optimal patient and family language for care and when patients desire interpretation can be complex.

In the literature there are several studies that include perspectives from clinical staff on the use of interpretation and communicating with families with LOE.^15–17^ There is less information about the patient and family perspective regarding determining language for care and the use of professional interpretation. In one qualitative study, Spanish-speaking families reported a lack of access to interpretation and experiences with providers overestimating their understanding of English.^18^ In another survey study, patients with LOE reported high satisfaction with the receipt of any professional interpretation with the favored modality being via remote video, then in person, and then by phone.^19^ To our knowledge, there are no studies related to the caregiver perspective on determining language for care and the use of professional interpretation during pediatric emergency care. The objective of this study was to describe the perspectives of caregivers with LOE in the pediatric ED about how best to determine language for care and utilize professional interpretation using a survey tool at or near PED discharge.

## Methods

This was a cross-sectional survey study using a convenience sample of patient caregivers with LOE at the time of discharge from a large academic PED. Between April and November 2021, caregivers of patients with LOE in the ED were approached by research staff using professional interpretation near the time of ED discharge. Language for care was determined using the standard 2-step process at ED check-in: *“What is your language for care today?”* and if the answer is a language other than English, the staff member calls a professional interpreter and asks, *“Can we provide free interpretation?”* The ED was in year 5 of an ongoing quality improvement initiative to increase interpretation. During the study period, approximately 82% of patients with LOE received interpretation during the encounter, and interpretation events (when remote) were 0.9 per hour, measured by connecting vendor invoices and in-person interpreter orders to patient encounters.^20^

Research coordinators monitored the ED tracking board for patients with LOE as identified by the standard ED process and approached caregiver(s) for consent to participate via telephone while still in the ED. The survey tool (Supplementary Appendix SA1) was developed by authors and members of the multi-disciplinary quality improvement team in the ED working to increase interpretation. It was piloted with staff in the ED, research coordinators, and caregivers with LOE in the ED prior to its use. Surveys were completed either on site near the time of PED discharge or within the following week by phone using professional interpretation or with a certified bilingual staff member. At the time of the study, the team included staff members with certification for Spanish and Vietnamese, thus a version of the survey was translated into these languages and reviewed by the bilingual staff to ensure accuracy, as opposed to using sight translation. The survey began with informed consent and a teach-back question to ensure good comprehension prior to continuing. Research staff asked the survey questions, and then entered the answers as well as patient identification, age, ED-assigned acuity level by emergency severity index (ESI), and language into REDCap.^21^

The goal was to include a representative sample of families with different languages for care. To achieve this, we prioritized approaching families by language to represent the most common languages in our ED population: Spanish, Somali, Russian, Vietnamese, Cantonese, Mandarin, Amharic, Oromo, and Tigrinya. We reviewed enrollment every 2 weeks during the study period to ensure a representative and diverse convenience sample.

Descriptive statistics were used to characterize the results, using R version 4.2. As this was a descriptive study, no inferential statistics were used. This study was approved by the hospital institutional review board.

## Results

### Participants

A total of 154 participants were approached and 49 (32%) completed the survey. An additional 14 consented to participate but either did not have sufficient time prior to discharge or were not available by phone after discharge to complete the survey. Among completed surveys, there were 15 unique languages (Table 1). These languages represented the overall PED population at the time, except for Spanish, which was slightly under-represented at 47% of our sample and 63% of the overall PED population during the study period. Most patients (47%) were assigned a moderate visit acuity level 3 using the Emergency Severity Index (ESI), 31% were lower acuity ESI level 4, and 22% were higher acuity level 2. Patient ages were 0–16 years with a median of 3 years.

**Table 1.** Languages Represented among Survey Participants

Language for care	*n* (%)
Spanish	23 (47%)
Somali	4 (8.2%)
Mandarin	3 (6.1%)
Oromo	3 (6.1%)
Amharic	2 (4.1%)
Arabic	2 (4.1%)
Cantonese	2 (4.1%)
Tigrinya	2 (4.1%)
Vietnamese	2 (4.1%)
Brazilian Portuguese	1 (2.0%)
French	1 (2.0%)
Korean	1 (2.0%)
Mongolian	1 (2.0%)
Portuguese	1 (2.0%)
Urdu	1 (2.0%)

### Reported Language Proficiency

All patients had indicated LOE at ED check in, but their reported levels of proficiency varied. There were 3 questions about English proficiency: speaking, understanding, and understanding medical conversations. Twenty percent reported proficiency in speaking English, 28% in understanding English, and 20% in understanding medical conversations. When asked if caregivers wanted a hospital interpreter for today’s visit, 86% said yes, but 12% said no. Of those who said no, 4 indicated they were comfortable enough with English, 3 said they brought a family member to interpret, and 1 was concerned about delays in care or cost. None reported wanting the patient to interpret.

### Desire for Interpretation during ED Visit

Most participants reported desiring interpretation for different steps of the ED visit, although there was some variation (Table 2). Nearly all caregivers reported having received “the right amount” of interpretation during their visit, with 1 reporting they wanted less interpretation.

**Table 2.** Interpretation at Different Stages of an ED Encounter

Step of ED encounter	Desire for interpretation	Reported receipt of interpretation
ED Check in	30/49 (61%)	27/49 (55%)
First conversation with doctor	44/49 (90%)	45/49 (92%)
Nursing updates	34/49 (69%)	35/49 (71%)
Medication administration	23/31 (74%)	22/22 (100%)
Discussion of results/next steps	41/49 (84%)	41/49 (84%)

### Identifying Accurate Language for Care

Most participants wanted to be asked about language at ED check in (80%) or when the doctor or nurse entered the room (78%). A smaller majority (71%) said to consult their medical record and always use interpretation for them. Seventy-three percent of participants reported wanting the ability to initiate access to the hospital interpreter themselves during the visit. Caregivers were less supportive of leaving a remote interpreter unit connected in the room between ED staff interactions, a practice some in our ED had advocated for because it decreases sign in time if multiple clinicians are entering the room in close succession. When asked the single best method of these options, the largest proportion (47%) answered to ask at ED check in, the next most frequent response was to provide family-initiated interpreter access.

### Factors Associated with Desire for Interpretation

Participants reported there could be differing interpretation needs among caregivers in their families; 39% (19) reported that another caregiver for the same patient may want a different amount of interpretation. They also reported a possible dependence on their child’s presenting illness; 59% said desire for interpretation depended on how serious the child’s sickness was. Finally, survey participants overall reported good comprehension of the discharge diagnosis and next steps, with 95% saying they understood well or very well.

## Discussion

In this descriptive analysis from a pediatric ED, we report the results from a survey of patient caregivers from a variety of different language backgrounds on determining language for care and interpretation practices.

Determining the optimal language for care for patients and families during an ED encounter can be challenging. A previous study has shown that asking a single question about language preference did not correlate well with the demonstrated ability to speak or understand English.^13^ In our survey, families who had indicated LOE through our 2-step check in process still reported differing levels of English comprehension, with approximately 20% reporting high proficiency in medical conversations. This indicates that there is not complete agreement between language for care as determined during the 2-step check-in process, preferred language, and reported proficiency. Health care professionals must be careful to ascertain what is best for each family and situation, and not make assumptions based on the provider’s assessment of a family’s English proficiency. Families may want interpretation in another language even when their English comprehension is high. Given this can be a complicated question, we also explored caregiver perspectives on how best to determine their language for care. Participants indicated the best methods would be asking about their language for care at ED check-in, which is frequently already done in EDs, but also giving them the ability to access professional interpretation themselves. The latter would be a promising shift away from the typical power dynamic of health care staff controlling access to interpretation and would be novel in most settings. Family access to interpretation will require piloting to ensure it does not introduce any additional barriers. For some families, it might be too much of a burden in a stressful and unfamiliar health care setting to also be responsible for accessing interpretation. Family-driven access may also inadvertently make hospital staff feel less responsible for the provision of interpretation and decrease interpreter use. We believe it is a direction worth pursuing that will also require careful exploration prior to implementation.

The use of interpretation in clinical settings is often reported in the literature as “any” or “none” during the patient encounter. However, this oversimplifies the many opportunities to communicate with families with LOE during their visit. In the pediatric ED, it has been shown that interpretation use during procedures, medication administration, and discharge may be low.^22^ In our study, we asked families with LOE if they wanted interpretation at different stages in the visit. Nearly all caregivers wanted interpretation during their first interaction with the doctor or nurse and when results were explained. All caregivers wanted interpretation during medication administration and reported receiving it; however, this is one area where interpretation was infrequently used in previous studies. A smaller majority of caregivers wanted interpretation immediately at ED check in or with every nurse check-in. This highlights how important professional interpretation use is at every single point in the ED visit and for every type of communication with staff. It is important to note, however, that there is some heterogeneity as to when caregivers want interpretation, underscoring some of the complexity in these interactions.

Fifty-nine percent of caregivers indicated the perceived severity of their ED visit affected their desire for interpretation. It has been shown that providers also make value judgements for “getting by” without interpretation depending on the perceived importance of the conversation they plan to have with the patient.^23^ However, it is unlikely that either the patient’s family or the providers can accurately predict the nature of every clinical interaction, again highlighting the importance of using professional interpretation with all types of conversations.

### Limitations

This study had several limitations. It was designed to be descriptive, and the numbers are relatively low so we cannot determine patterns in responses according to language backgrounds. The survey was either administered by certified bilingual staff or using professional interpretation. The latter may have biased participant responses, particularly when asking about the quality or amount of interpretation used during the visit. We did have bilingual research staff (Spanish, Vietnamese) doing the enrollment whenever possible. The survey required 40–60 min to complete, which was a barrier to enrolling caregivers and completing the questions; this contributed to our relatively low rate of completion. Caregivers were approached near the end of the ED visit and for some there was not sufficient time to complete the survey questions. The caregivers surveyed did not include ESI 1 or ESI 5, the most and least acute visits, respectively, due to the limitations of a survey during active resuscitation or within the scope of a brief ED visit. We did not collect data on whether there was one or more caregivers answering the questions, although we did ask about the interpretation needs of various family members. There is a risk for desirability bias as patients were interviewed by hospital staff, although research staff administering the surveys used a standard script so that caregivers knew they were not part of the care team and participation was optional. There may also have been inaccuracies in the interpretation of the survey, although the teach-back question was designed to determine adequate comprehension.

## Conclusion

In this descriptive study in the pediatric ED, caregivers from a variety of language backgrounds completed a survey regarding their perspectives on determining language for care and the use of professional interpretation. Although participants indicated they wanted interpretation at ED check in, approximately 20% also reported a high level of English proficiency in medical conversations. Most caregivers wanted interpretation at all points during the ED encounter, although there was some heterogeneity. Families wanted to be asked about language interpretation at ED check in but were also interested in having their own ability to access professional interpretation when desired. Families also reported that different caregivers for the patient would likely need differing amounts of interpretation. Our findings overall support that providing high quality equitable and language-concordant care can be complex. There is a need for more patient and family perspectives, especially in some of the more nuanced aspects of providing medical interpretation such as determining the optimal language for care at each patient encounter. Given the layers of complexity, it is important for the health care team to err towards providing more interpretation and ensuring it is available for all types of interactions in the PED. Our next steps are to pilot adding an option for direct family-initiated access to interpreter services, to continue to explore ways to tailor interpreter access and use to individual family preference and need.

## Health Equity Implications

Utilizing professional interpretation for all communication during PED encounters is crucial for ensuring equitable care for families and patients with different language backgrounds. Our findings provide insight into family and caregiver perspectives on interpretation at one PED and offer an exciting new direction of piloting family-imitated access to interpretation.

## Supplementary Material

Supplementary Appendix SA1

## Abbreviations Used

ED: Emergency department
ESI: Emergency severity index
LOE: Language for care other than English
PED: Pediatric emergency department
PI: Professional interpretation
