# Supplementary material for: Patient Caregiver Perspectives on Accessing Language Interpretation in a Pediatric Emergency Department
Source: Health Equity. 2024 Sep 12;8(1):648–53. doi: 10.1089/heq.2024.0010 (PMC11464821; doi:10.1089/heq.2024.0010)
Supplement: Supplementary Appendix SA1 [file heq.2024.0010_suppl_datas1.pdf]

<u>Patient Survey for patients and families with LOE identified at ED check-in</u>

Please answer one of the following:

How well do you speak English?
Very well   Well   Not well   Not at all

How well do you usually understand English?
Very well   Well   Not well   Not at all

How well do you usually understand your doctor or nurse in English?
Very well   Well   Not well   Not at all

The next few questions are about interpretation. When we say "hospital interpreter" we mean interpretation by a hospital interpreter in-person, on a video unit, or over a phone call.

Did you want a hospital interpreter during your recent/current emergency room visit?
Yes   No   I'm not sure

If no, why not? I will read a list of possible reasons, please answer yes/no

- Family member can interpret (Yes/no)
- Patient can interpret (Yes/no)
- I'm comfortable enough in English (Yes/no)
- It takes too long, I thought it might delay care (Yes/no)
- I'm concerned about cost (Yes/no)
- Is there another different reason you did not want interpretation? (free text)

At what points during your ER visit did you **want** hospital interpretation and at which points did you **receive** hospital interpretation? Think about each part of the ER visit as I read it to you; for each, I will ask if you wanted interpretation and if you received it.

Think about checking in to the emergency room:
- Did you want interpretation then? Yes/No
- Did you receive interpretation then? Yes/No

Think about the first talk with the doctor:
- Did you want interpretation then? Yes/No
- Did you receive interpretation then? Yes/No

Think about any updates you received about your child's care during the visit:
- Did you want interpretation then? Yes/No
- Did you receive interpretation then? Yes/No

Think about every time the nurse came into the room to check your child:
- Did you want interpretation then? Yes/No

Did you receive interpretation then? Yes/No

Did your child receive any medications during the visit? (Yes/No)
(if yes, then proceed, otherwise skip)
Did you want interpretation then? Yes/No
Did you receive interpretation then? Yes/No

Think about when the doctor explained any test or examination results and the plan for what to do next:
Did you want interpretation then? Yes/No
Did you receive interpretation then? Yes/No

During your recent visit, how did you receive hospital interpretation? I will read a list, and you can say yes or no to each of them.
Phone Yes/No/ I'm not sure
Video Yes/No/ I'm not sure
In person Yes/No/ I'm not sure
I did not receive any hospital interpretation

Think about how much interpretation you received on your most recent visit: Did you want:
More/Less
Or was the amount just right?

How was the quality of the hospital interpretation when it was provided?
Very good Good Okay Not good Bad

How satisfied were you with the way we asked if you wanted hospital interpretation today?
Very satisfied Somewhat satisfied Not satisfied at all

Now I am going to read a list of ways that we might know if you want interpretation or not. For each one, tell me if you think we should definitely do it, maybe do it, or not do it.
Ask when you check in to the ED: Yes definitely/Yes, maybe/ No
Ask each time you talk with the nurse/doctor: Yes definitely/Yes, maybe/ No
Always provide interpretation if your preferred language is in the record: Yes definitely/Yes, maybe/ No
Leave the interpreter on in the room during your visit for staff to use when necessary: Yes definitely/Yes, maybe/ No
Make it so you are able to access the hospital interpreter yourself when you want it: Yes definitely/Yes, maybe/ No

Of the ones we just went over, which way do you think it best? I can repeat the options if you would like me to

Do you have other ideas of how and when we should ask you if you want interpretation? (free text)

Are there other caregivers in your family (for example, another parent or grandparent) who might want a different amount of hospital interpretation than you do?
Yes No Unsure

Does whether or not you want interpretation depend on how sick your child is or how severe the problem is?
Yes, definitely Yes, maybe Not really No

How well do you understand your child's diagnosis from your visit?
Very well Well Not well Not at all

How well do you understand what happened in the ER and your next steps?
Very well Well Not well Not at all

Would it be ok if we contact you again to ask you more questions about your experience?
